# Metabolomics and genetics of reproductive bud development in *Ficus carica* var. *sativa* (edible fig) and in *Ficus carica* var. *caprificus* (caprifig): similarities and differences

**DOI:** 10.3389/fpls.2023.1192350

**Published:** 2023-06-08

**Authors:** Ilaria Marcotuli, Manuela Mandrone, Ilaria Chiocchio, Ferruccio Poli, Agata Gadaleta, Giuseppe Ferrara

**Affiliations:** ^1^ Department of Soil, Plant and Food Sciences, University of Bari “Aldo Moro”, Bari, Italy; ^2^ Dipartimento di Farmacia e Biotecnologie, Alma Mater Studiorum - Università di Bologna, Bologna, Italy

**Keywords:** brebas, main crop, metabolome, sucrose, fruit production

## Abstract

In figs, reproductive biology comprises cultivars requiring or not pollination, with female trees (edible fig) and male trees (caprifig) bearing different types of fruits. Metabolomic and genetic studies may clarify bud differentiation mechanisms behind the different fruits. We used a targeted metabolomic analysis and genetic investigation through RNA sequence and candidate gene investigation to perform a deep analysis of buds of two fig cultivars, ‘Petrelli’ (San Pedro type) and ‘Dottato’ (Common type), and one caprifig. In this work, proton nuclear magnetic resonance (^1^H NMR-based metabolomics) has been used to analyze and compare buds of the caprifig and the two fig cultivars collected at different times of the season. Metabolomic data of buds collected on the caprifig, ‘Petrelli’, and ‘Dottato’ were treated individually, building three separate orthogonal partial least squared (OPLS) models, using the “y” variable as the sampling time to allow the identification of the correlations among metabolomic profiles of buds. The sampling times revealed different patterns between caprifig and the two edible fig cultivars. A significant amount of glucose and fructose was found in ‘Petrelli’, differently from ‘Dottato’, in the buds in June, suggesting that these sugars not only are used by the ripening brebas of ‘Petrelli’ but also are directed toward the developing buds on the current year shoot for either a main crop (fruit in the current season) or a breba (fruit in the successive season). Genetic characterization through the RNA-seq of buds and comparison with the literature allowed the identification of 473 downregulated genes, with 22 only in profichi, and 391 upregulated genes, with 21 only in mammoni.

## 1 Introduction

*Ficus* is the largest genus in the Moraceae family, showing great diversity in morphology (leaves, inflorescences, and fruits), breeding systems (monoecious and dioecious), and pollination types (Berg, 1989; Bronstein and McKey, 1989; Berg and Corner, 2005; Chawla et al., 2012). The *Ficus* genus is approximately 75 million years old (Cruaud et al., 2012), and many fig species are native to tropical regions with only a few ones in warm and temperate areas. The *Ficus* genus can be classified into six subgenera, namely, two monoecious subgenera (*Urostigma* and *Pharmacosycea*) and four functionally dioecious subgenera (*Sycomorus*, *Ficus*, *Synoecia*, and *Sycidium*). Approximately half of the total *Ficus* species are monoecious (Berg, 1989), such as *Ficus sycomorus* (Galil and Eisikowitch, 1968) and *Ficus aurea* (Hossaert-McKey and Bronstein, 2001), and the others are gynodioecious, such as *Ficus carica* (2n = 26) (Condit, 1947). The dioecious *Ficus* species are generally less synchronized than monoecious species such as *F. aurea* (Hossaert-McKey and Bronstein, 2001). *Ficus* species have diverse growth habits, with an important role in tropical, subtropical, and Mediterranean ecosystems to feed with their fruits the seed dispersers of many species (Shanahan and Compton, 2001; Harrison, 2005; Cottee-Jones et al., 2016). The *Ficus* species most important for commercial cultivation is *F. carica* L., called the common (edible) fig, which is gynodioecious and includes several cultivars.

**Figure 1:**
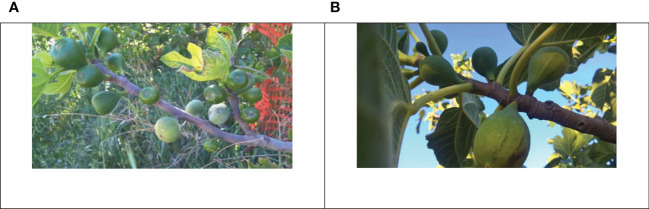
A caprifig **(A)** with mamme (pale green, at the bottom of the 1-year-old shoot) and profichi (vivid green, at the top of the 1-year-old shoot) and an edible fig **(B)** with brebas (big fruits on 1-year-old shoot) and figs (main crop, small growing fruits on the current year shoot).

The female (edible) fig can produce one to three fruits: 1) brebas (parthenocarpic), at the end of spring to the beginning of summer on the 1-year-old shoot; 2) figs (main crop), in summer on the current-year shoot; and 3) a late crop at the end of summer to the beginning of autumn on the current-year shoot. In some cultivars, this latter crop can be the only main fruit (figs, late crop). The male fig (caprifig) can have three fruits similar to the female fig: 1) the profichi in spring to the beginning of summer on the 1-year-old shoot, 2) the mammoni in summer–autumn on the current-year shoot, and 3) the mamme during autumn–winter on the current-year shoot (over-wintering fruits). According to their pollination behavior, the numerous edible fig cultivars have been grouped into three main groups: 1) the Common type, with persistent fruits; 2) the Smyrna type, necessarily requiring pollination; and 3) the San Pedro type, requiring or not pollination (Storey, 1955; Ferrara et al., 2016; Marcotuli et al., 2020). Brebas are always produced parthenocarpically, whereas the main and late fruits (figs) are either parthenocarpic or produced after pollination depending on the cultivar. Considering only the pollination behavior, figs could be classified into two groups: 1) requiring pollination to set fruits or 2) not requiring pollination since they are able to set fruits parthenocarpically (persistent fruits).

In the common fig, the female trees (*F. carica* var. *sativa* L.) are often pollinated, or “caprificated”, by the male trees (*F. carica* var. *caprificus* L.), called “caprifig” to set the fig (main crop). The wasps (*Blastophaga psenes*) can carry pollen from male flowers, located close to the ostiole in the inedible profichi fruits (hermaphrodite, with male and short-styled female flowers) borne on caprifig trees, to the long-styled female flowers of the edible figs (unisexual with pistillate flowers in the main crop, or “fig”) driven by olfactory sense toward the flavors emitted by the fruits.

However, either in the common fig or in the caprifig, all the buds are developed on the same shoot (Condit, 1947; Marcotuli et al., 2020). The apical/lateral bud, normally a mixed one, develops a shoot carrying axillary fruits in summer-autumn (i.e., figs) and/or flower buds developing in the successive season (i.e., brebas). In the common fig, the basal/middle portion of the shoot is generally occupied by the main and/or the late crop (when occurring) and the distal by the breba crop, whereas in the caprifig, the basal portion is occupied by the mammoni (when occurring), the middle by the more abundant mamme, and the distal by the profichi (Lama et al., 2022). This localization of the fruits occurs when all the types of fruits are present and usually only two types of fruits are present (breba/fig and profichi/mamme) (**Figure 1**).

It is unknown why some flower buds on the current year shoot may develop in summer for the figs (main crop), in autumn for the late crop, or the successive year for the breba crop since they are all present on the same shoot of the current season. It seems that the evolution of each bud on the nodes of the 1-year shoot is already predetermined, but it is unclear when or how this predetermination is defined. New techniques and devices can help to clarify some of these unsolved questions.

Sweetness is perhaps the main indicator of fruit quality in figs and is determined by the soluble sugar concentration. Ripe fig fruits are very rich in sugars, and sugar accumulation is a developmental process. Sugar levels remain low during the first phases of development, and concentrations increase considerably during the final stages of ripening, until harvest. The role of sugar in bud differentiation has been investigated in a recent study (Lama et al., 2022).

NMR is increasingly being used for plant metabolomic profiling due to its capability to analyze complex traits. NMR-based metabolomics, which is often approached through untargeted analysis and whose data are handled with multivariate analysis techniques, has already been applied successfully in several areas of research. In particular, in plant science, NMR metabolomics has been used to facilitate the identification of active compounds in medicinal plants (Mandrone et al., 2018; Salomé-Abarca et al., 2020), for taxonomical and phylogenetic studies (Maggio et al., 2016; Cheng et al., 2020), for food, fruit, and botanical quality control and fraud detection (Rizzuti et al., 2015; Callao and Ruisánchez, 2018; Sherman et al., 2020; Mandrone et al., 2021a), and for plant physiology and plant–environment interaction studies (Mandrone et al., 2021b; Mandrone et al., 2022). It is, in fact, a fast, robust, reliable, and non-destructive technique, with quick and simple sample preparation, and, importantly, it allows to obtain quantitative information due to the linear relationship between the integral of a resonance peak and the respective metabolite concentration. Moreover, NMR has no dependence on the ionization of the metabolites. However, NMR profiling has limitations due to the sensitivity of the technique itself; thus, it is optimal to acquire an overview of the most abundant metabolites in an extract but not suitable to detect metabolites present at very low concentrations in the mixture. Recently, the availability of a high-quality fig reference genome provided an important resource to genetic improvement and breeding programs, and Mori et al. (2017) released a preliminary genome sequence of a Japanese cultivar of *F. carica*, ‘Horaishi’, which was affected by the typical deficiencies of short-read genome assemblies (Veeckman et al., 2016; Low et al., 2019). Usai et al. (2020) recently reported a high-quality genome reference for the cultivar ‘Dottato’. Genetic analysis with RNA-seq provides a useful instrument to understand complex and massive biological processes, by data integration and processing at multiple levels of biological systems (Fabres et al., 2017). Transcriptomic data may provide new insights for fig breeding and postharvest management.

The present work aimed to understand the process of bud differentiation at two nodes (3 and 5) of two fig cultivars (Common and San Pedro type) and one caprifig by using both a metabolomic, using ^1^H NMR, and a genetic investigation, with RNA sequences analysis and candidate gene investigation in order to understand what makes a bud develop into a fig in the current year or enter into dormancy and develop into a breba in the following season.

## 2 Results

### 2.1 ^1^H NMR-based metabolomic analysis

**Figure 2:**
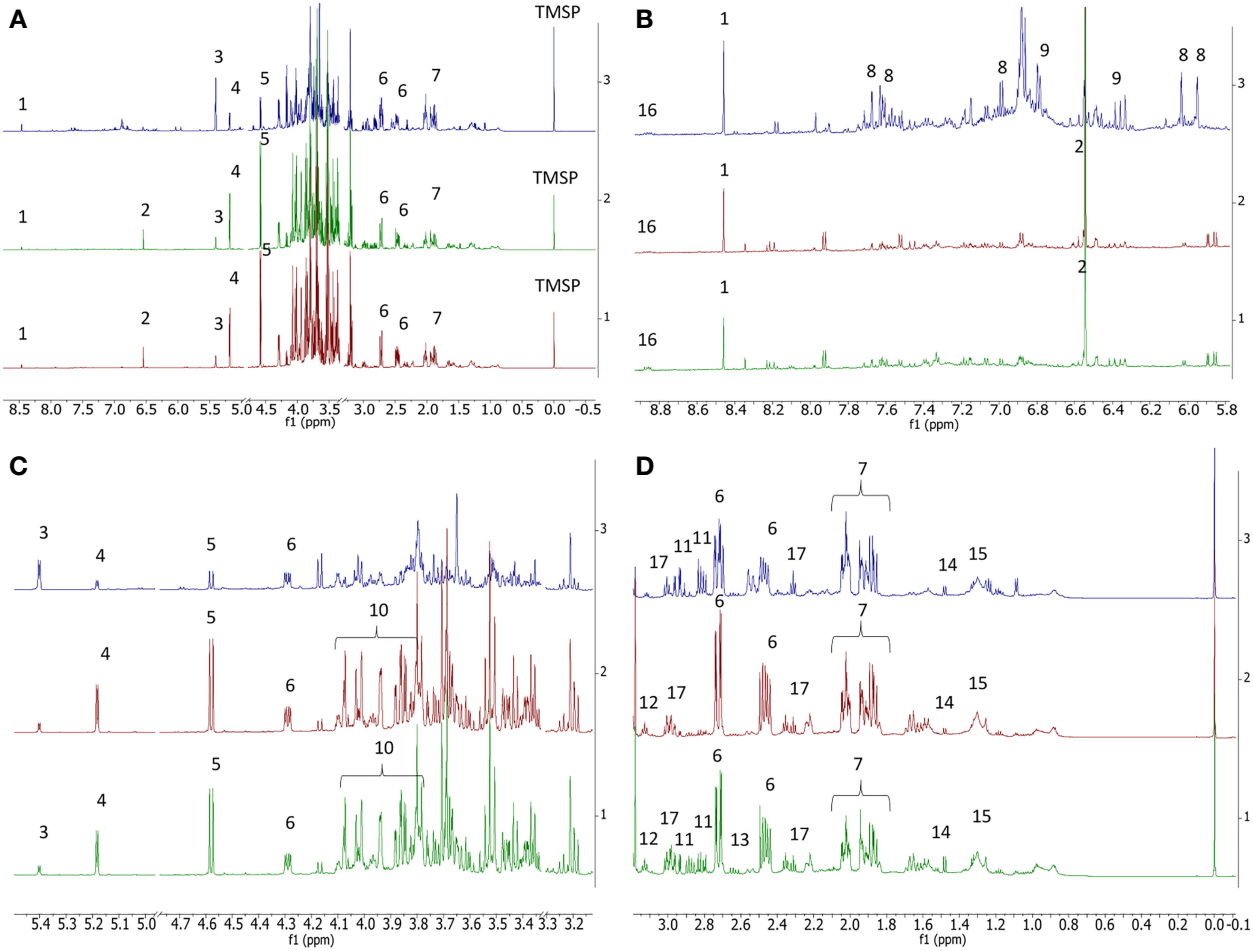
Comparison between ^1^H NMR profiles of caprifig buds (blue trace), ‘Dottato’ buds (green trace), and ‘Petrelli’ buds (red trace) collected in June. **(A)** Full spectra. **(B)** Enlarged region between δ 5.95 and 9. **(C)** Enlarged region between δ 5.5 and 3.1. **(D)** Enlarged region between δ −0.1 and 3.1. 1 = formic acid, 2 = fumaric acid, 3 = sucrose, 4 = α-glucose, 5 = β-glucose, 6 = malic acid, 7 = quinic acid, 8 = rutin, 9 = chlorogenic acid, 10 = fructose, 11 = asparagine, 12 = α-ketoglutarate, 13 = aspartic acid, 14 = alanine, 15 = lipids, 16 = trigonelline, and 17 = GABA. Spectral references are given in **Table S1**.

This study represents the first NMR-based metabolomic analysis on both fig and caprifig buds. In the June samples, the presence of several metabolites was evident from the spectra, including organic acids, such as formic acid, fumaric acid, malic acid, α-ketoglutarate, quinic acid, and GABA, sugars, namely, α- and β-glucose, sucrose, and fructose, and amino acids, such as alanine, asparagine, and aspartic acid. However, citric acid was not detected (**Figure 2**). Moreover, secondary metabolites such as trigonelline, chlorogenic acid, and rutin were also detected. The presence of rutin and chlorogenic acid was further confirmed by liquid chromatography–mass spectrometry (LC-MS), finding negative ions at *m*/*z* 609.152 and 353.088, respectively, and positive ions at *m*/*z* 633.142 and 377.084 corresponding to [M+ Na] adducts. At the same time of the season, the flower buds of both fig cultivars and the caprifig showed similar spectra with regard to the metabolomic analysis but with some important remarks (**Figure 2**). After proper data processing, the complete dataset obtained was analyzed by principal component analysis (PCA). This unsupervised model was performed to acquire a first overview of sample distribution based on metabolomic profile. This analysis showed the complexity of the dataset; in fact, no clustering was evident (**Supplementary Figure S1**), likely due to the highly diversified nature of samples: buds collected at two nodes, two fig cultivars, and a caprifig and at different times during the season (June to October). Consequently, metabolomic data of buds collected on caprifig and the two fig cultivars were treated individually, building three separate orthogonal partial least squared (OPLS) models, using the sampling time as the “y” variable.

Through these analyses, it was possible to find correlations between metabolomic profiles of buds and sampling times, which revealed different patterns between caprifig and the two edible fig cultivars.

**Figure 3:**
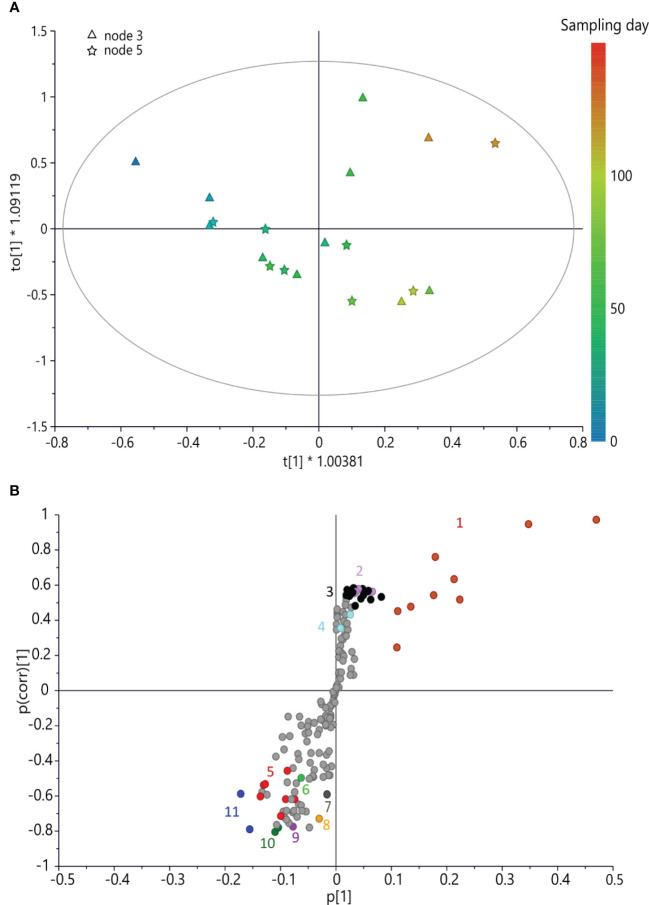
**(A)** OPLS score scatter plot built using bucketed ^1^H NMR spectra of ‘Dottato’ buds as the *x*-axis and sampling day as the *y*-axis; the sampling time is highlighted by the color gradient, from blue (which indicates the earliest collections) to red (for the latest). **(B)** S-plot of the OPLS model, highlighting the *x*-axis (spectral signals) highly correlated with harvesting time (*y*-axis). 1 = sucrose, 2 = rutin, 3 = spectral region from δ 6.05 to 7.05 (aromatic region), 4 = chlorogenic acid, 5 = quinic acid, 6 = alanine, 7 = trigonelline, 8 = formic acid, 9 = GABA, 10 = spectral region from δ 1.61 to 1.69, and 11 = spectral region from δ 3.17 to 3.25. OPLS, orthogonal partial least squared.

In the case of ‘Dottato’, the model (**Figure 2**) was fitted by two components (*R* ^2^ *x*(cum) = 72.6%, *R* ^2^y(cum) = 86.2%, and *Q* ^2^(cum) = 80.9%). Its predictability was confirmed by *R* ^2^ *y*(cum) and *Q* ^2^(cum) obtained by permutation tests, which were 86.2% and 80.9%, respectively; the intercept on the *y*-axis of the *Q*-line was −0.446; and the intercept of the *R*-line was 0.21. CV-ANOVA *F* and *p* were 14.797 and 6.24 × 10^−5^, respectively. In the early stage (June) of bud development (both nodes) of ‘Dottato’, a higher concentration of trigonelline, formic acid, quinic acid, alanine, GABA, and other unknown metabolites (responsible for signals at δ 3.2 and 1.65) was found. In late summer/beginning of autumn, these compounds were gradually decreasing in both nodes, whereas sucrose and aromatic compounds were increasing because of the quiescence/dormancy of the buds. Rutin and chlorogenic acid resulted in two of the most important aromatic compounds present in the extracts; however, other unidentified aromatic metabolites (spectral signals from δ 6.7–6.9) increased along the sampling times (**Figure 3**).

**Figure 4:**
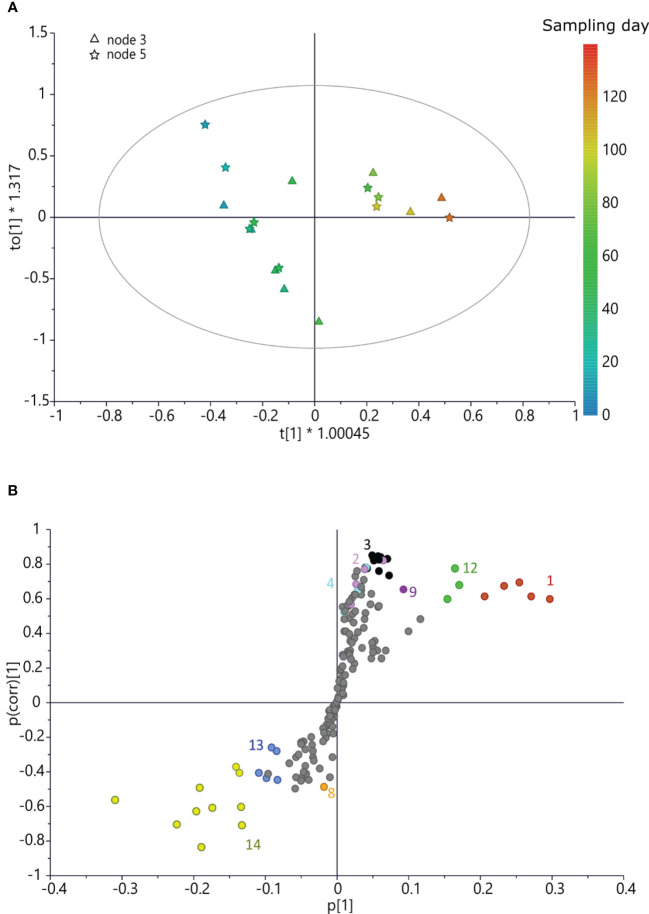
**(A)** OPLS score scatter plot built using bucketed ^1^H NMR spectra of ‘Petrelli’ buds as the *x*-axis and sampling day as the *y*-axis; the sampling time is highlighted by the color gradient, from blue (which indicates the earliest collections) to red (for the latest). **(B)** S-plot of the OPLS model, highlighting the *x*-axis (spectral signals) highly correlated with harvesting time (*y*-axis). 1 = sucrose, 2 = rutin, 3 = spectral region from δ 6.05 to 7.05 (aromatic region), 4 = chlorogenic acid, 8 = formic acid, 9 = GABA, 12 = malic acid, 13 = glucose, and 14 = fructose. OPLS, orthogonal partial least squared.

As for ‘Dottato’, also in ‘Petrelli’ at three- and five-node buds, both sucrose and aromatic compounds (including rutin and chlorogenic acid) increased in the late sampling dates, i.e., at the end of the growing season (**Figure 4**). However, in this cultivar, malic acid was also more abundant in late summer. GABA also increased at this stage of development in ‘Petrelli’, following an opposite pattern compared to ‘Dottato’.

However, together with formic acid, a high concentration of glucose and fructose characterized the buds at the early stage of development. The OPLS model on ‘Petrelli’ samples was fitted by three components (*R* ^2^ *x*(cum) = 85.9%, *R* ^2^ *y*(cum) = 94%, and Q^2^(cum) = 71.6%). The permutation test gave *R* ^2^ *y*(cum) = 94% and Q^2^(cum) = 71.6%; the intercept on the *y*-axis of the Q-line was −0.727; and the intercept of the R-line was 0.68. CV-ANOVA F and *p* were 3.60 and 0.03, respectively (**Figure 4**).

**Figure 5:**
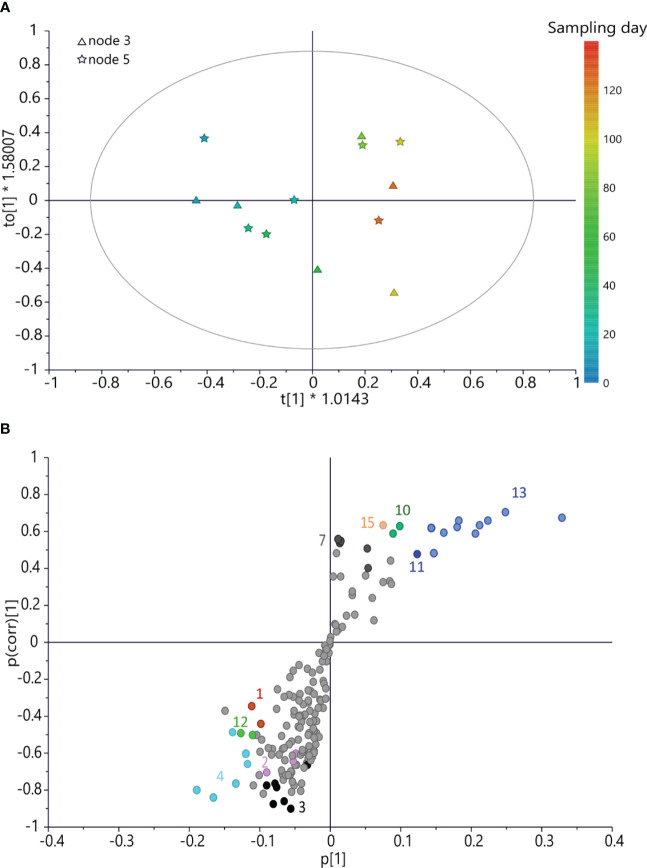
**(A)** OPLS score scatter plot built using bucketed ^1^H NMR spectra of “caprifig” buds as the *x*-axis and sampling day as the *y*-axis; the sampling time is highlighted by the color gradient, from blue (which indicates the earliest collections) to red (for the latest). **(B)** S-plot of the OPLS model, highlighting the *x*-axis (spectral signals) highly correlated with harvesting time (*y*-axis). 1 = sucrose, 2 = rutin, 3 = spectral region from δ 6.05 to 7.05 (aromatic region), 4 = chlorogenic acid, 7 = trigonelline, 10 = spectral bins from δ 1.61 to 1.69, 11 = spectral bins from δ 3.17 to 3.25, 12 = malic acid, 13 = glucose, and 15 = lipids. OPLS, orthogonal partial least squared.

A further OPLS model (**Figure 5**) was built using the x variable using bucketed ^1^H NMR spectra of buds collected on the caprifig and harvesting time for the *y*-axis. This model was fitted by three components (*R* ^2^ *x*(cum) = 74.3%, *R* ^2^ *y*(cum) = 88.1%, and Q^2^(cum) = 21.5%). The permutation test gave *R* ^2^ *y*(cum) = 88% and Q^2^(cum) = 21%, the intercept on the *y*-axis of the Q-line was −0.342, and the intercept of the R-line was 0.54. CV-ANOVA F and *p* were 0.38 and 0.84, respectively. This analysis provided interesting information. In fact, different from the two fig cultivars, caprifig buds yielded higher levels of aromatic compounds (such as rutin and chlorogenic acid) and sucrose at the early stage of development, and also malic acid was more abundant. As the sampling time progresses, lipids increase along with glucose and trigonelline. Notably, glucose and trigonelline were involved also in the development of ‘Petrelli’ and ‘Dottato’ buds, respectively, but followed an opposite pattern compared to “caprifig”, since in ‘Petrelli’ and ‘Dottato’, they were more abundant in the early sampling times.

In all the developed models, generally, the metabolomic profiles of buds collected at nodes 3 and 5 were quite similar between them with some specific differences in the pattern of some compounds.

### 2.2 Differential gene expression analysis in caprifig

Among the total expressed genes, 473 were downregulated, with 22 only in profichi, and 391 were upregulated, with 21 only in mammoni (**Table 1**). Looking at the function of upregulated genes, 225 followed the molecular function category, 54 genes belonged to the cellular component, and 112 belonged to the biological process, while among the downregulated genes, 151 coded for the biological process, 61 for the cellular component, and 261 for molecular function (**Figure S2**).

**Table 1:** Total number of loci identified in our genotypes, number of loci expressed in caprifig and in two time points, and regulation obtained from the RNA-seq analysis.

	Loci expressed in caprifig	Loci expressed in profichi	Loci expressed in mammoni
Total number	23,098	22,088	21,031
Downregulated	473	22	–
Upregulated	391	–	21

Comparing the number of transcripts detected in profichi and mammoni from caprifig with the same fig buds from ‘Petrelli’ and ‘Dottato’, two *F. carica* cultivars belonging to the San Pedro and Common type, respectively (previously studied with RNA-seq analysis as reported in Marcotuli et al., 2020), the number of expressed loci was different; in particular, caprifig showed a higher number of transcripts in profichi (22,088) and the lowest number in mammoni (21,031) when compared to the two fig cultivars (**Figure S3**). Within the 22,088 and 21,031 loci expressed in caprifig in profichi and mammoni, respectively, 2,254 were unique for caprifig with 36 singles for profichi and 12 for mammoni.

Considering that among the upregulated and downregulated genes, there were 22 upregulated specifically for profichi and 21 downregulated unique for mammoni, the analysis of these specific genes was carried out to determine the role in the caprifig bud development.

The evaluation of the upregulated genes underlined how six genes were responsible for biological processes and molecular function, two for cellular components, and 14 for molecular function (**Table 2**). Analyzing the downregulated genes, seven corresponded to biological processes, three to cellular components, and 11 to molecular function (**Table 3**).

**Table 2:** List of loci upregulated and expressed only in profichi buds (from caprifig tree) was obtained with the RNA-seq analysis.

GO category	GO description	Locus	Locus ‘Dottato’	Genes ‘Dottato’	Chr	Expression level	KEGG annotation	NCBI annotation
biological_process	Fatty acid biosynthetic process	s00042g04609	CM019741.1	scaffold4994_size7080_1_6190_F_g8478.t1	3	59.27	Lipoxygenase	Linoleate 13*S*-lipoxygenase
	Response to light stimulus	s00693g22683	CM019742.1	scaffold46213_size1701_246_1645_F_g34891.t1	4	3.48	–	Gibberellin 20 oxidase
	Regulation of systemic acquired resistance	s00207g12592	CM019748.1	scaffold25821_size4231_2377_3511_R_g25563.t1	10	5.18	–	–
	Malate transport	s00025g03163	CM019750.1	scaffold31761_size2380_1_2259_F_g28761.t1	12	1.51	–	Aluminum-activated malate transporter
	Pigment biosynthetic process	s00026g03301	CM019739.1	scaffold10590_size4980_283_2371_R_g14493.t1	1	3.94	Polyphenol oxidase	Polyphenol oxidase
	Recognition of pollen	s00095g08016	CM019750.1	scaffold16001_size3915_1886_3417_F_g19050.t1	12	0.33		G-type lectin S-receptor-like serine/threonine-protein kinase
cellular_component	Origin recognition complex	s00903g25124	CM019747.1	scaffold13243_size4394_1_4221_R_g16822.t1	9	1.01	Origin recognition complex subunit 5	Origin of replication complex subunit 5
	Intracellular	augustus_masked-BDEM01000280.1-processed-gene-1.7	CM019751.1	scaffold322_size15895_5397_7851_R_g991.t1	13	1.64	–	–
molecular_function	RNA polymerase II transcription factor activity, sequence-specific DNA binding	s00038g04288	VYVB01000457.1	scaffold3676_size7976_7043_7871_R_g6729.t1	–	3.91	Transcription factor MYB, plant	MYB transcription factor
	Catalytic activity	augustus_masked-BDEM01000023.1-processed-gene-3.11	CM019750.1	scaffold9629_size6575_659_6538_F_g13580.t1	12	1.89	–	Anthocyanidin reductase-like
	RNA-directed DNA polymerase activity	s00241g13648	CM019749.1	scaffold105_size19749_4726_13077_F_g364.t1	11	0.40	–	–
	Acid phosphatase activity	s00541g20410	CM019739.1	scaffold33766_size2261_1_691_R_g29715.t1	1	94.48	–	Acid phosphatase
	Carbonate dehydratase activity	s00006g01019	CM019743.1	scaffold66917_size1159_1_710_F_g41455.t1	5	19.63	Carbonic anhydrase	Carbonic anhydrase
	Polygalacturonase activity	s00391g17456	CM019742.1	scaffold69640_size1106_1_821_R_g42133.t1	4	11.35	Polygalacturonase	Polygalacturonase
	Structural constituent of cell wall	augustus_masked-BDEM01000491.1-processed-gene-0.0	CM019741.1	scaffold26864_size2722_1596_2722_F_g26160.t1	3	6.21	–	Extensin-2-like
	Cysteine-type peptidase activity	s00368g16967	CM019740.1	scaffold16048_size3908_1266_3908_F_g19086.t1	2	0.10	–	Senescence-specific cysteine protease
	Hydrolase activity, acting on ester bonds	augustus_masked-BDEM01000490.1-processed-gene-0.6	CM019745.1	scaffold32446_size2338_1_1251_R_g29086.t1	7	0.51	–	GDSL esterase/lipase
	Hydrolase activity, acting on glycosyl bonds	s12649g33674	CM019740.1	scaffold15871_size3937_1026_3845_F_g18949.t1	2	13.89	–	Heparanase-like protein
	Ligase activity	s00006g01157	CM019743.1	scaffold29_size24094_17182_19171_R_g125.t1	5	2.00	–	E3 ubiquitin-protein ligase
	Carbohydrate binding	s01131g27036	CM019751.1	scaffold27470_size2674_1086_2164_F_g26500.t1	13	132.87	–	–
	Polysaccharide binding	s01280g27982	CM019750.1	scaffold40297_size1938_1_1151_R_g32576.t1	12	2.51	–	Wall-associated receptor kinase-like
	NADP binding	s00058g05819	CM019748.1	scaffold11747_size4701_1_2111_R_g15510.t1	10	18.60	–	Indole-3-pyruvate monooxygenase

The genes were associated with biological processes, cellular components, and molecular function using the GO database; the chromosome location was assigned through the ‘Dottato’ genome database, while the putative gene function was assigned using the KEGG and NCBI databases.

GO, Gene Ontology; KEGG, Kyoto Encyclopedia of Genes and Genomes; NCBI, National Center for Biotechnology Information.

**Table 3:** List of loci downregulated and expressed only in mammoni buds (from caprifig tree) obtained with the RNA-seq analysis.

GO category	GO description	Locus	Locus ‘Dottato’	Genes ‘Dottato’	Chr	Expression level	KEGG annotation	NCBI annotation
biological_process	Signal peptide processing	s00652g22144	CM019741.1	scaffold11116_size4854_1_4781_R_g14964.t1	3	0.46	–	Signal peptidase complex
	Metabolic process	augustus_masked-BDEM01000001.1-processed-gene-12.10	VYVB01000409.1	scaffold8926_size9877_5197_6441_R_g12884.t1	–	2.41	7-Deoxyloganetin glucosyltransferase	7-Deoxyloganetin glucosyltransferase
	Response to biotic stimulus	s00232g13405	CM019746.1	scaffold1415_size10907_236_1251_R_g3238.t1	8	0.23	–	Pathogenesis-related protein
	Embryo development ending in seed dormancy	s00025g03175	CM019750.1	scaffold33232_size2291_1456_2259_F_g29464.t1	12	2.37		Late embryogenesis abundant protein
	Cell wall modification	s00266g14365	CM019749.1	scaffold33026_size2304_1_1441_R_g29371.t1	11	3.15	Pectinesterase	Pectinesterase
	Lignin catabolic process	s00107g08572	CM019742.1	scaffold19235_size3477_1752_3101_R_g21415.t1	4	2.46	Laccase	Laccase-15-like
	Positive regulation of response to water deprivation	s00033g03881	CM019739.1	scaffold20836_size3292_1356_2847_F_g22474.t1	1	82.83	–	Late embryogenesis abundant protein
cellular_ component	Cell wall	s00172g11363	CM019746.1	scaffold190_size17850_11955_14011_R_g624.t1	8	1.46	–	Late embryogenesis abundant protein D-34-like
	Lipid particle	s00117g09066	CM019744.1	scaffold10586_size4980_2886_3789_F_g14489.t1	6	3.10	–	Oleosin-like protein
	Monolayer-surrounded lipid storage body	s00177g11527	CM019744.1	scaffold13563_size4332_1905_2871_R_g17092.t1	6	1.36	–	Oleosin-like protein
molecular_function	d-Arabinono-1,4-lactone oxidase activity	s00046g04937	CM019745.1	scaffold51567_size1529_1_1529_R_g36765.t1	7	71.01	–	Probable l-gulonolactone oxidase
	DNA-directed RNA polymerase activity	s04066g31473	CM019743.1	scaffold10696_size4953_895_2491_R_g14581.t1	5	19.46	–	–
	Diacylglycerol *O*-acyltransferase activity	augustus_masked-BDEM01003719.1-processed-gene-0.0	VYVB01000496.1	scaffold46893_size1677_1_1622_F_g35145.t1	–	3.28	–	*O*-Acyltransferase WSD1-like
	*N*,*N*-Dimethylaniline monooxygenase activity	s00527g20163	CM019740.1	scaffold4834_size7176_1_2484_F_g8278.t1	2	4.45	–	Probable flavin-containing monooxygenase
	Serine-type endopeptidase inhibitor activity	s00002g00355	CM019748.1	scaffold24423_size2926_2116_2521_R_g24725.t1	10	10.55	–	Proteinase inhibitor
	UDP-*N*-Acetylmuramate dehydrogenase activity	s00046g04936	CM019745.1	scaffold43128_size1818_1226_1818_F_g33704.t1	7	22.85	–	Probable l-gulonolactone oxidase
	Transferase activity, transferring hexosyl groups	s00001g00185	VYVB01000409.1	scaffold8926_size9877_7551_9631_R_g12885.t1	–	0.37	7-Deoxyloganetin glucosyltransferase	7-Deoxyloganetin glucosyltransferase-like
	Manganese ion binding	s00318g15730	CM019741.1	scaffold6918_size6155_3146_4501_R_g10715.t1	3	0.16	–	Germin-like protein subfamily 1
	Nutrient reservoir activity	s00002g00296	CM019748.1	scaffold52422_size1504_1_1504_F_g37041.t1	10	341.93	–	11S globulin precursor isoform
	Protein heterodimerization activity	s00032g03793	CM019739.1	scaffold1820_size10111_484_1388_F_g3955.t1	1	1.36	Nuclear transcription Y subunit beta	Nuclear transcription factor Y
	Peroxiredoxin activity	s00558g20689	CM019749.1	scaffold48362_size1626_1_451_R_g35683.t1	11	5.75	Peroxiredoxin 6, 1-Cys peroxiredoxin	1-Cys peroxiredoxin

The genes were associated with biological processes, cellular components, and molecular function using the GO database; the chromosome location was assigned through the ‘Dottato’ genome database, while the putative gene function was assigned using the KEGG and NCBI databases.

GO, Gene Ontology; KEGG, Kyoto Encyclopedia of Genes and Genomes; NCBI, National Center for Biotechnology Information.

### 2.3 Transcriptome analysis of flowering genes during bud differentiation and development

To define the genes potentially involved in the caprifig bud development process, we considered the unique genes upregulated and downregulated in profichi and mammoni as reported in **Tables 2**, **3**.

Considering all the genes expressed and upregulated in profichi, we detected oxidase genes, in particular the linoleate 13*S*-lipoxygenase, that are involved in a number of diverse aspects of plant physiology including growth and development and that catalyze hydroperoxidation of lipids containing a *cis*,*cis*-1,4-pentadiene structure (Park et al., 2020). We also detected gibberellin 20 oxidase, which is involved in the promotion of the floral transition, fertility, and stem elongation (Li et al., 2021), polyphenol oxidase, which oxidizes monophenols and diphenols in the presence of molecular oxygen and could be activated during the first weeks of storage (McLarin and Leung, 2020), and anthocyanidin reductase-like, which catalyzes the NADPH-dependent conversion of anthocyanidins into flavonoids (Tang et al., 2009).

Additionally, the aluminum-activated malate transporter, which is involved in GABA transport, and the indole-3-pyruvate monooxygenase, which is involved in auxin biosynthesis genes, were identified. Genes implicated in cell cycle control and cell expansion during plant development were also reported and in particular the G-type lectin S-receptor-like serine/threonine-protein kinase, the origin of replication complex subunit 5, MYB transcription factor and wall-associated receptor kinase-like, extensin-2-like, senescence-specific cysteine protease, GDSL esterase/lipase, heparanase-like protein, and E3 ubiquitin-protein ligase. Finally, three hydrolase genes were found represented by acid phosphatase, carbonic anhydrase, and polygalacturonase.

Considering the genes downregulated in mammoni, some of the genes were reported in more than one locus such as the 7-deoxyloganetin glucosyltransferase (two loci), which is most expressed in anthers, and plays a major role in cell homeostasis (Qu et al., 2021); late embryogenesis abundant protein (three loci), which induces the maturation drying process of embryo development correlates with the acquisition of desiccation tolerance (Hoekstra et al., 2001); oleosin-like protein (two loci), which is involved in species recognition by discriminating between compatible and incompatible pollen (Schein et al., 2004); and probable l-gulonolactone oxidase (two loci), which is associated with the endoplasmic reticulum membrane and synthesizes the oxidation of l-gulonolactone to l-ascorbic acid (Linster and Van Schaftingen, 2007). Even in this case, genes for plant development were identified, in particular the signal peptidase complex, which acts in the translocation of polypeptide chains across the endoplasmic reticulum membrane (Linster and Van Schaftingen, 2007); the 11S globulin precursor isoform, which is involved in seed storage protein (Hsiao et al., 2006); the nuclear transcription factor Y that acts in gametogenesis, embryogenesis, seed development, flowering time regulation, and abscisic acid signaling (Mu et al., 2013); pectinesterase, which facilitates plant cell wall modification and subsequent breakdown (Suzuki et al., 2013); and the *O*-acyltransferase WSD1-like, which catalyzes the condensation of fatty alcohol and a fatty acyl-Coenzyme A (acyl-CoA) (De la Fuente Cantó et al., 2018). The analysis of the downregulated genes in figs (main crop) highlighted the presence of genes involved in the plant defense response, such as probable flavin-containing monooxygenase gene, 1-Cys peroxiredoxin, pathogenesis-related protein, and proteinase inhibitor.

**Figure 6:**
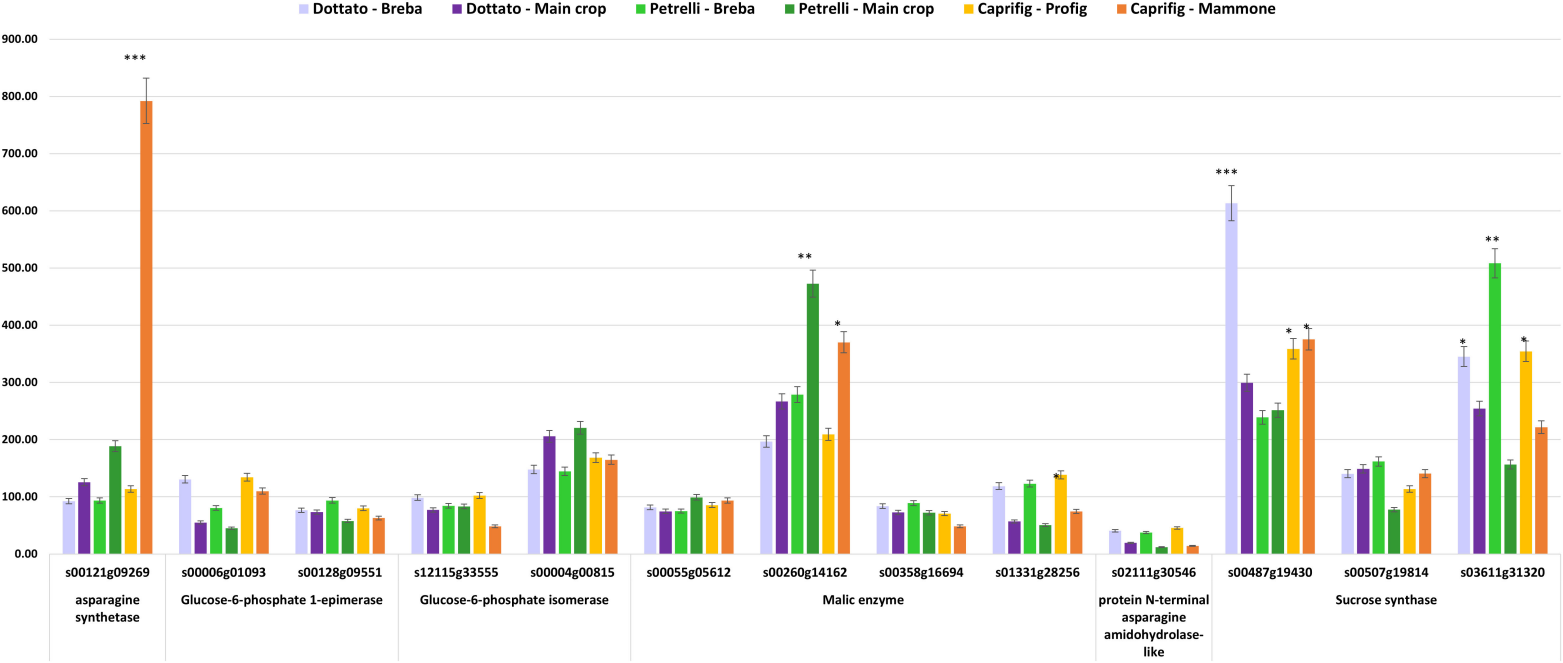
Quantitative expression data (reported as FPKM values) of genes correlated to metabolites detected by ^1^H NMR breba/profig and fig (main crop)/mammoni of caprifig, ‘Dottato’, and ‘Petrelli’. Asterisks on the bars indicate datasets significantly different according to ANOVA (*, **, and *** correspond to *p* < 0.05, *p* < 0.01, and *p* < 0.001, respectively).

In order to correlate the metabolomic data with the genetic ones, genes involved in the biosynthetic pathway of the detected metabolites identified through the ^1^H NMR-based analysis were investigated among all the expressed genes in both cultivars, ‘Dottato’ and ‘Petrelli’, and in the caprifig. We were able to identify 13 loci and six genes with an expression level, reported as RPKM value, higher than 50, and correlated to the metabolites detected (**Table S2**). In particular, the analysis allowed the identification of: asparagine synthetase (one locus), which showed a peak of expression in mammoni, while similar values were reported for profichi and ‘Dottato’ and ‘Petrelli’ breba and fig (main crop) (**Figure 6**) (Lama et al., 2022); glucose-6-phosphate 1-epimerase (two loci), which converts α-glucose in β-glucose, andshowed higher expression in brebas compared to figs (main crops) in both fig cultivars, and with the caprifig showing a general higher level compared to the two cultivars; glucose-6-phosphate isomerase (one locus), which converts glucose in fructose, and is highly expressed in ‘Dottato’ and ‘Petrelli’ figs (main crops) (Lama et al., 2022); malic enzyme (four loci), which showed different values in relation of the locus considered (**Figure 6**); protein N-terminal asparagine amidohydrolase-like (one locus), which converts asparagine into aspartic acid and showed higher expression levels in brebas/profichi of all the genotypes; and sucrose synthase (three loci), which showed higher values in ‘Dottato’ and ‘Petrelli’ brebas and for one locus also in caprifig (**Figure 6**).

## 3 Discussion

In monoecious species of *Ficus*, male and female flowers grow on the same tree, and self-pollination occurs, although cross-pollination may partly occur, which could help to reduce inbreeding depression.

Metabolomic analysis revealed differences in the two fig cultivars and the caprifig, and values were also different in the two nodes, thus indicating a correlation between metabolomic profiles of buds and sampling and position times, which showed a significantly different pattern between caprifig and the two edible fig varieties.

It was surprising that citric acid, an important and abundant organic acid of fig fruits (Jafari et al., 2022), was not detected at the budding level, according to the metabolomic analyses, whereas minor organic acids such as quinic and fumaric acid were present in the buds.

In ‘Dottato’, trigonelline, formic acid, quinic acid, alanine, and GABA were more abundant in buds sampled in June and decreasing in late summer samples, whereas sucrose and aromatic compounds were high in June and decrease in late summer. Trigonelline is synthesized from nicotinic acid, which is a catabolite of pyridine nucleotides, and accumulates in seeds of legumes and coffee (Ashihara et al., 2015); several hypotheses have been proposed on the physiological functions of trigonelline in plants (Ashihara et al., 2015). A massive increase in trigonelline content was found during the early stages of maturation in the pericarp of coffee fruits (Ashihara et al., 2015). The role of trigonelline in ‘Dottato’ buds may be related to the storage of nicotinic acid or a compatible solute to face stress conditions during the growing stage in summer, i.e., drought, or to contribute indirectly to the formation of desirable flavor products of the fruit to attract the wasp for the pollination.

The detection of quinic acid, a side product of the shikimate pathway regulated by the activity of quinate dehydrogenase, at this stage of bud development, suggested a behavior similar to the kiwifruit’s accumulation of quinic acid, although with different concentrations (Jiang et al., 2020). However, quinic acid was also detected in the sap of another fig species, *Ficus dubia*, and was suggested that such phenolic derivatives either are from the shikimate pathway or are used for biosynthesis of chlorogenic acid (Chansriniyom et al., 2021), which was detected in large amounts in the successive samplings. Quinic acid was also found in the leaves of Portuguese *F. carica* cultivars ‘Pingo de mel’ and ‘Branca Tradicional’ (Chansriniyom et al., 2021). Chlorogenic acid and rutin have been recently found in different tissues of several fig cultivars, from fruits (peel and pulp) to leaves (Teruel-Andreu et al., 2021). Chlorogenic acids and rutin have been generally extracted from the leaves more than present in fruits or buds (Mahmoudi and Chawla, 2022) and are possibly translocated from leaves to developing buds.

In both fig cultivars, at 3 and 5-node buds, a significant increase of both sucrose and aromatic compounds was detected in the late sampling dates. However, sucrose is generally low in the fruits throughout development and maturation (Vemmos et al., 2013), but sucrose increases during fruit development, in stages I and II, and then decreases after ripening. In this case, the increase of sucrose in the fruit buds of both ‘Dottato’ and ‘Petrelli’ may suggest the preparation of the buds for dormancy, and sugars could be used, such as in peach (Santiago-Mejía et al., 2018) and in sweet cherry (Fadón et al., 2018), to support the differentiation of the various tissues of a flower bud, i.e., xylem, anthers, and ovary. Moreover, sucrose would be used in the successive season for the development of possible breba fruits in spring. Sucrose could be used to sustain the first development stages of the brebas prior to the leaf’s full functionality for the production of photosynthates. In fact, carbohydrate reserves act as key agents in dormant buds, and thus they accumulate before the end of the growing season and are utilized following bud break (Mohamed et al., 2012). Carbohydrate reserves are also used as an indicator of bud break in many fruit trees (Kaufmann and Blanke, 2017). In a recent study conducted on the fig cultivar ‘Sabz’, soluble sugars decreased during bud break, bringing about new leaf development and shoot growth in spring (Sedaghat et al., 2022). These carbohydrates were possibly transformed into structural forms, resulting in new organ formation (leaves and stems) and an increase in plant volume (Sedaghat et al., 2022). Soluble sugars are also partly lost through the energetic process of transpiration (Spann et al., 2008).

The presence of sucrose in both ‘Dottato’ and ‘Petrelli’ fruit buds may also suggest that both varieties are physiologically prone to yield such type of early fruit, although brebas of ‘Dottato’ generally drop and are seldom produced. Differently from sucrose, glucose and fructose contents increase during fruit growth and ripening (Sedaghat and Rahemi, 2018). The major soluble sugars in fig leaves and fruits are sucrose, glucose, and fructose, with the latter being more abundant. Sucrose is always higher than other sugars in the leaves (Gaaliche et al., 2022) since it is the end product of photosynthesis and the primary sugar transported in the phloem. Sucrose in the fig buds could be either transported to or synthesized in the buds, which can photosynthesize when they have chlorophyll. Differently from ‘Dottato’, the presence of glucose and fructose in ‘Petrelli’, already in the buds at early sampling times (June), may indicate that these sugars are not only used by the ripening brebas of this cultivar but are also used by the developing buds on the current-year shoot (flower, mixed, and vegetative buds).

However, in ‘Petrelli’, malic acid is also more abundant in late summer. Pande and Akoh (2010) identified five organic acids in fresh figs: malic, citric, oxalic, ascorbic, and succinic acids. Citric and malic acids are the primary organic acids found in the fruits of figs (Pereira et al., 2017). Shikimic and fumaric acids have also been identified in the peel and pulp of figs (Oliveira et al., 2009). Chlorogenic acid, which is abundant at the end of the growing season in ‘Petrelli’ (**Figure 3**), is reported to be among the major individual phenolic compounds in whole fig fruit (Talon et al., 1990).

GABA was found to increase in the last sampling times in ‘Petrelli’, following an opposite pattern compared to ‘Dottato’. When considering an important crop such as citrus, GABA is more active or mostly functioning during citrus fruit ripening (Cercós et al., 2006). In particular, a reduction of approximately 42% of GABA was determined by comparing young and older citrus fruits (Cercós et al., 2006). The different GABA contents in the fruit buds of ‘Dottato’ and ‘Petrelli’ may indicate the different evolutions of the buds toward brebas, fig, or even latent buds.

Many genomic tools are available for the ‘Dottato’ cultivar, including a haplotype-phased genome sequence (Usai et al., 2020) and a leaf transcriptome (Fattorini et al., 2021), but no information is available on metabolomics in buds. Previous studies showed that the ‘Dottato’ transcriptome is very different from that of another fig cultivar, such as ‘Horaishi’ sequenced by Mori et al. (2017).

We found that sugar accumulation is a key factor also in bud development. Key genes involved in sugar content variability were previously identified, and their expression was compared between unripe fruits and ripe fruits of cultivars ‘Dottato’ and ‘Brogiotto’ (Fattorini et al., 2021). In addition, in the present work, the sucrose synthase (three loci) showed higher values in Dottato and ‘Petrelli’ buds and for one locus in caprifig. Fattorini et al. (2021) also found in ‘Dottato’ an increased expression of a gene encoding a sucrose synthase, SUSY1, in ripe fruits, while SUSY6 showed a reduced expression. The high content of sucrose at the end of samplings in ‘Dottato’ and ‘Petrelli’ and glucose in caprifig may suggest either a possible use during the dormant stage of the buds or that fruit buds accumulated sugars (as the fruits) without the growth of the fruit. In the latter case, it seems as if the fruit bud is not completely activated in the season to develop a fruit and will overwinter. The fruit buds on the current season shoot seem all ready to develop fruit, with the increase of sugars, but other factors, i.e., hormonal control, make some of them develop in fruits and others to overwinter.

Cui et al. (2019) also identified key enzymes and genes (sucrose synthase 2) involved in sucrose accumulation, and blasting the protein sequence in public databases, it was found that upregulated sucrose synthase gene had high similarity to both *Vitis vinifera* (88.7%) and *Citrus unshiu* sucrose synthase (84.4%).

The key role of sugar accumulation was confirmed by the positive correlation found between sugar metabolomic data with the genetic ones; in fact, in the present work, 13 loci and six genes with an expression level higher than 50 RPKM value correlated with the metabolites detected. The glucose-6-phosphate 1-epimerase locus, which converts α-glucose into β-glucose, showed higher expression in brebas compared to figs (main crops) in both cultivars and with the caprifig showing a generally higher level compared to the two cultivars. Glucose-6-phosphate isomerase (one locus), which converts glucose into fructose, was highly expressed in ‘Dottato’ and ‘Petrelli’ figs.

## 4 Materials and methods

### 4.1 Plant materials and bud sampling

Two fig cultivars grown at the fig repository equipped with environmental and soil sensors (Torres et al., 2017) at the “P. Martucci” experimental station in Valenzano (Bari) belonging to the University of Bari “Aldo Moro”, Department of Soil, Plant and Food Science—Tree Fruit Unit, and a caprifig tree located in a private orchard not far from the fig repository, were used in this work. The two cultivars were 1) ‘Dottato’ (also known as ‘Kadota’) of the Common type and 2) ‘Petrelli’ of the San Pedro type. In summer 2021, starting from June to October, buds at two different nodes (3 and 5 from the basal part) of the current year shoots of both the two cultivars and the caprifig were sampled at an interval of approximately 10 days starting from June until the beginning of October (with a total of 51 samples), placed in a paper bag, and rapidly transported in a portable fridge to the lab for storage in a −80°C refrigerator. For caprifig samples, RNA-seq and candidate genes analysis were also conducted on fruit buds harvested at two different times, corresponding to profichi and mammoni.

### 4.2 ^1^H NMR-based metabolomic analysis

Samples were grounded in a mortar using liquid nitrogen. For each node of the two fig cultivars and the caprifig, 30 mg of freeze-dried and powdered buds underwent ultrasound-assisted extraction for 20 min (TransSonic TP 690, Elma, Germany) using 1 ml of a mixture (1:1) of phosphate buffer (0.1 M; pH 6.0) in H_2_O-*d*_2_ (containing 0.01% trimethyl silyl propionic acid sodium salt (TMSP)) and MeOH-*d*_4_. The extraction solvents were selected based on previous metabolomics studies (Johri and Konar, 1956; Kim and Verpoorte, 2010; Kim et al., 2010; Jafari et al., 2022), which reported that a mixture of methanol and aqueous phosphate buffer, pH 6.0 (1:1), provided a good overview of both secondary and primary metabolites, giving the best extraction conditions to obtain a broad spectrum of compounds from plant samples. Since there was no previous research on the NMR-based metabolomics of fig buds, we decided to use the most generic extraction protocol for broad-spectrum extraction in NMR-metabolomics. After this procedure, samples were centrifuged for 10 min (17,000 ×*g*), and then 600 μl of supernatant was transferred into NMR tubes and analyzed. ^1^H NMR spectra were recorded at 25°C on a Varian Inova instrument (equipped with a reverse triple-resonance probe) operating at a ^1^H NMR frequency of 600.13 MHz, and H_2_O-*d*_2_ was used as an internal lock. Each ^1^H NMR spectrum consisted of 256 scans (corresponding to 16 min) with a relaxation delay (RD) of 2 s, acquisition time of 0.707 s, and spectral width of 9,595.8 Hz (corresponding to δ 16.0). A presaturation sequence (PRESAT) was used to suppress the residual water signal at δ 4.83 (power = −6 dB, presaturation delay 2 s). The analysis of ^1^H NMR profiles of extracts was performed based on an in-house library (for plant metabolites since online libraries are for human metabolites) and in comparison with the literature, and each identified metabolite was also semi-quantified using the internal standard TMSP.

### 4.3 Multivariate data analysis

For multivariate analysis, data were Pareto scaled and subjected to multivariate data analysis (PCA and OPLS) through SIMCA P+ software (v. 15.0, Umetrics, Sweden). OPLS models were built using the harvesting time for the *y*-axis, which was calculated as the number of days from the first day of harvesting (ranging from day 1 to day 124). The obtained OPLS models were evaluated by the goodness of fit (*R* ^2^ *x* (cum) and *R* ^2^ *y*(cum)) and goodness of prediction (Q^2^(cum)), together with the parameters given by cross-validation tests: permutation test (performed using 200 permutations) and CV-ANOVA.

### 4.4 UPLC-qTOF-MS data acquisition

This further analysis was required to confirm the structure of rutin and chlorogenic acid, which were present at low concentrations, and some of their diagnostic ^1^H NMR signals overlapped with those of other metabolites.

Flower bud hydroalcoholic extracts at a concentration of 0.5 mg/ml were analyzed using a Xevo G2-XS QTof system (Waters, Milford, MA, USA) equipped with a polar C18 analytical column (Luna Omega, 100 × 3.0 mm, 3 µm particle size, Phenomenex, Torrance, CA, USA). The column was kept at 45°C, while the samples were kept at a constant temperature of 10°C. The mobile phases were H_2_O (A) and MeCN (B). The method and gradients used were the following: 95% A for 1 min followed by a gradient reaching 25% B in 2 min, 25% B was kept for 1 min, then the gradient reached 70% B in 3 min, 70% B was kept for 1 min, and then the gradient reached 5% B again in 20 s. The flow rate was 0.4 ml/min, and the injection volume was 2 µl.

Electrospray ionization in positive and negative modes was applied in the mass scan range of 50−1,200 *m*/*z*. Electrospray ionization (ESI) source conditions were as follows: capillary = 0.8 kV, cone = 40 V, source temperature = 120°C, desolvation temperature = 600°C, cone gas flow = 50 L/h, and desolvation gas flow = 1,000 L/h.

### 4.5 Total RNA extraction and differential gene expression analysis

Fruit buds from caprifig, harvested at two different times, June and July, were used for the extraction of total RNA according to the RNeasy Plant Mini Kit (QIAGEN^®^) instructions. In order to determine the standard deviation among replicates, at each stage, three different biological replicates were used, with three technical replicates. Considering that no variation was detected among the technical replicates, single samplings were mixed for the subsequent analysis. NanoDrop 2000 was used to check RNA quality and quantity (Thermo Scientific, Waltham, MA, USA) and samples were visualized on 1.5% agarose gel. RNA sequencing preparation and performance were carried out following the instruction reported by Marcotuli et al. (2020). Gene expression values in caprifig samples were quantified and considered statistically differentially expressed with a false discovery rate (FDR) value ≤0.05 as reported in the previous manuscript on fruit development in *F. carica* L (Marcotuli et al., 2020)..

The gene sequences of *F. carica* obtained with RNA-seq analysis (e-value threshold ≤E−10 and identity percentage higher than 80%) and differentially expressed in caprifig were assigned to biological process, cellular component, and molecular function through the Gene Ontology (GO) database, and the chromosome locations were identified using the locus ID GenBank (http://www.ncbi.nlm.nih.gov/Genbank/) and the Basic Local Alignment Search Tool (BLAST) (https://blast.ncbi.nlm.nih.gov/Blast.cgi) to find regions of similarity with the ‘Dottato’ genome sequence (Usai et al., 2020). Additionally, the Kyoto Encyclopedia of Genes and Genomes (KEGG) and National Center for Biotechnology Information (NCBI) databases were queried to define the putative biological pathways of the genes and to determine the role in fruit ripening at two different fruits (time points).

Additionally, expressed genes upregulated and downregulated were compared to ‘Petrelli’ and ‘Dottato’ genes previously analyzed and published (Lama et al., 2022).

## Data availability statement

The original contributions presented in the study are publicly available. This data can be found here: NCBI BioProject, accession PRJNA623468.

## Author contributions

Conceptualization: GF and AG. Methodology: IM and MM. Software: IM, MM, and IC. Validation: AG, FP, and MM. Formal analysis: IM and IC. Data curation: AG and FP. Writing—original draft preparation: IM, AG, GF, and MM. Writing—review and editing: IM, AG, GF, MM, and FP. Funding acquisition: GF and AG. All authors contributed to the article and approved the submitted version.
