# Supplementary material for: Metabolomics and genetics of reproductive bud development in Ficus carica var. sativa (edible fig) and in Ficus carica var. caprificus (caprifig): similarities and differences
Source: Front Plant Sci. 2023 Jun 8;14:1192350. doi: 10.3389/fpls.2023.1192350 (PMC10285451; doi:10.3389/fpls.2023.1192350)
Supplement: Supplementary file 1 [file DataSheet_1.docx]

***Supplementary Material***

**Metabolomics and genetics of reproductive bud development in *Ficus carica* var. *sativa* (edible fig) and in *Ficus carica* var. *caprificus* (caprifig): similarities and differences**

**Ilaria Marcotuli^1^, Manuela Mandrone^2^, Ilaria Chiocchio^2^, Ferruccio Poli^2^, Agata Gadaleta^1*^ and Giuseppe Ferrara^1*^**

1Department of Soil, Plant and Food Sciences, University of Bari “Aldo Moro”, via G. Amendola 165/A, 70126, Bari, Italy, ilaria.marcotuli@uniba.it, agata.gadaleta@uniba.it^*^, giuseppe.ferrara@uniba.it*.

^2^Alma Mater Studiorum - Università di Bologna, Dipartimento di Farmacia e Biotecnologie, Via Irnerio, 42 - 40126 Bologna, Italy, manuela.mandrone2@unibo.it, ilaria.chiocchio2@unibo.it, ferruccio.poli@unibo.it

1. ***Correspondence: giuseppe.ferrara@uniba.it; agata.gadaleta@uniba.it Tel.: +390805442979**
2. **Supplementary Figures and Tables**

**Table S1**. Diagnostic signals in ^1^H NMR profile of the detected metabolites. Chemical shifts, splitting pattern and coupling constants (Hz) are reported.

| Metabolite | diagnostic signals in ^1^H NMR profiles |
| --- | --- |
| alanine | 1.48 (d, J = 7.2 Hz) |
| α-ketoglutarate | 3.12 (t), |
| asparagine | 2.98 (dd) |
| aspartate | 2.95 (dd, J = 16.9, 4.1 Hz), 2.81 (dd, J = 16.9, 8.2 Hz) |
| chlorogenic acid | 7.50 (d, J = 15.9 Hz), 7.13 (d, J = 1.9 Hz), 7.07 (dd, J = 8.2, 1.9 Hz), 6.89 (d, J = 8.2 Hz), 6.37 (d, J = 15.9 Hz) 5.29 (m), 3.87 (dd, J = 8.8, 3.3 Hz), 2.23 (m), 2.10 (m) |
| fumaric acid | 6.53 (s) |
| formic acid | 8.46 (s) |
| fructose | 4.07 (m), 394 (m) |
| GABA | 3.00 (t, 7.2 Hz), 2.3 (t, 7.3 Hz) |
| *α*-glucose | 5.18 (d, J = 3.8 Hz) |
| *β*-glucose | 4.58 (d, J =7.8 Hz) |
| malic acid | 4.28 (dd, J = 9.2, 3.4 Hz), 2.71 (dd, J = 15.7, 3.5 Hz) |
| quinic acid | 1.93 (m), 1.90 (m), 1.86 (m) |
| rutin | 7.68 (d, J = 2.2 Hz), 7.63 (dd, J = 8.4, 2.2 Hz), 6.99 (d, J = 8.4 Hz), 6.04 (d, J = 2.2 Hz), 5.95 (d, J = 2.2 Hz) |
| sucrose | 5.40 (d, J = 3.8 Hz), δ 4.17 (d, J = 8.6 Hz) |
| trigonelline | 9.14 (s); 8.86 (m) |

**Table S2** Detailed data of genes correlated to metabolites detected by ^1^HNMR analysis in breba/profichi profig and main crop/mammoni of caprifig, ‘Dottato’ and ‘Petrelli’, with locus ID and gene expression profiling.

| **GO description** | **Locus** | **Expression RPKM** | | | | | |
| --- | --- | --- | --- | --- | --- | --- | --- |
|  |  | **Dottato - Breba** | **Dottato - Main crop** | **Petrelli - Breba** | **Petrelli - Main crop** | **Caprifig - Profig** | **Caprifig - Mammone** |
| *asparagine synthetase* | s00121g09269 | 92.36 | 125.60 | 93.42 | 188.60 | 113.70 | 792.03 |
| *Glucose-6-phosphate 1-epimerase* | s00006g01093 | 130.48 | 55.07 | 80.65 | 44.87 | 134.14 | 109.93 |
|  | s00128g09551 | 76.55 | 73.16 | 93.70 | 57.83 | 79.86 | 62.87 |
| *Glucose-6-phosphate isomerase* | s12115g33555 | 98.57 | 76.90 | 84.20 | 83.11 | 102.18 | 48.55 |
|  | s00004g00815 | 147.88 | 205.88 | 144.54 | 220.50 | 168.63 | 164.75 |
| *Malic enzyme* | s00055g05612 | 81.30 | 74.64 | 75.05 | 99.14 | 85.55 | 93.45 |
|  | s00260g14162 | 196.65 | 266.65 | 278.74 | 472.81 | 209.06 | 370.04 |
|  | s00358g16694 | 83.72 | 72.56 | 88.93 | 72.08 | 70.72 | 48.63 |
|  | s01331g28256 | 118.67 | 56.94 | 123.01 | 50.73 | 138.30 | 74.50 |
| *protein N-terminal asparagine amidohydrolase-like* | s02111g30546 | 40.61 | 19.42 | 37.75 | 12.01 | 45.49 | 14.23 |
| *Sucrose synthase* | s00487g19430 | 613.22 | 299.22 | 238.81 | 251.40 | 358.74 | 375.31 |
|  | s00507g19814 | 140.40 | 148.86 | 161.83 | 77.57 | 113.40 | 140.69 |
|  | s03611g31320 | 345.19 | 254.16 | 508.22 | 156.39 | 354.41 | 221.63 |


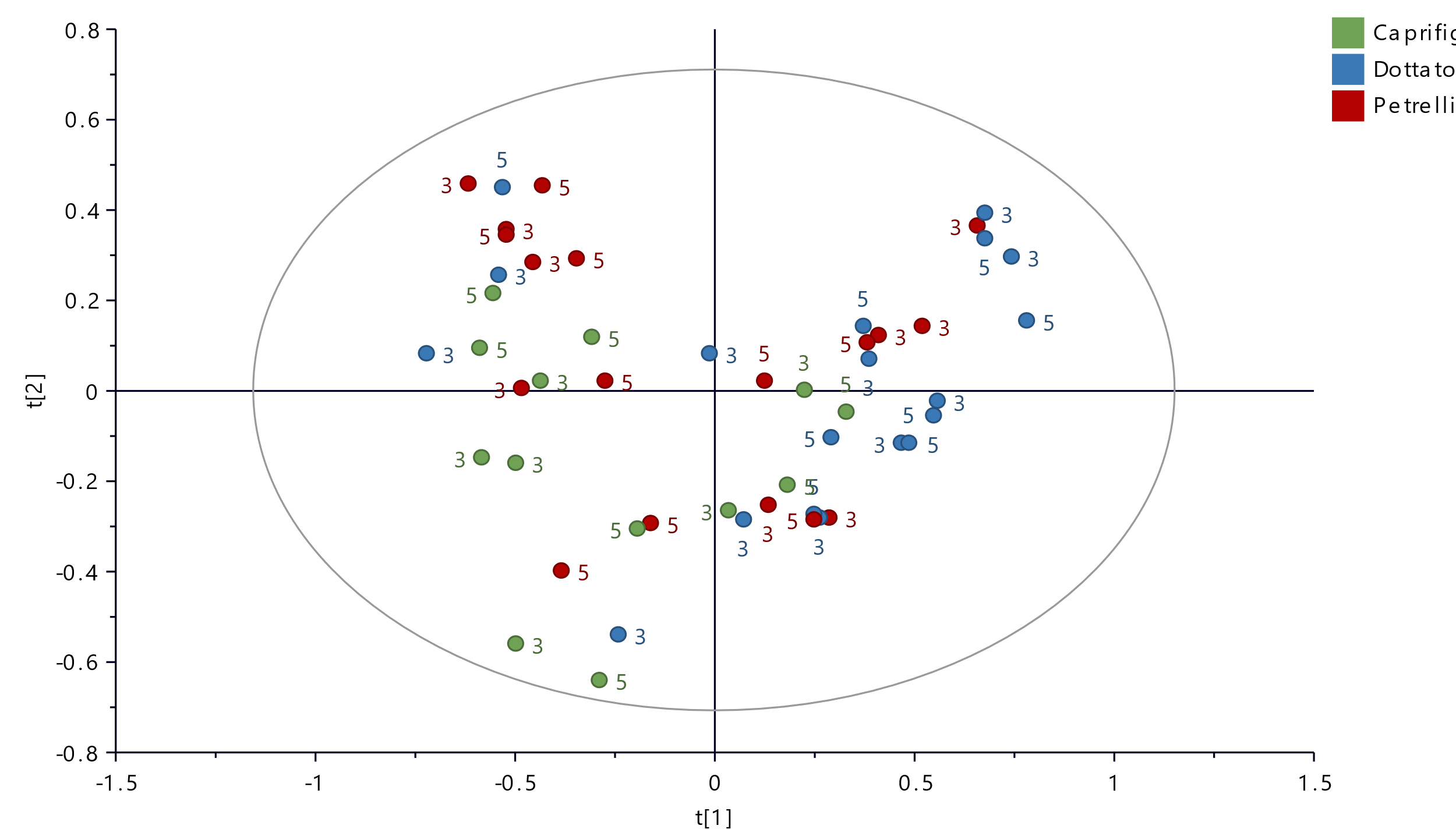


**Figure S1.** Principal Component Analysis score scatter plot obtained using the bucketed spectra of all the collected samples.


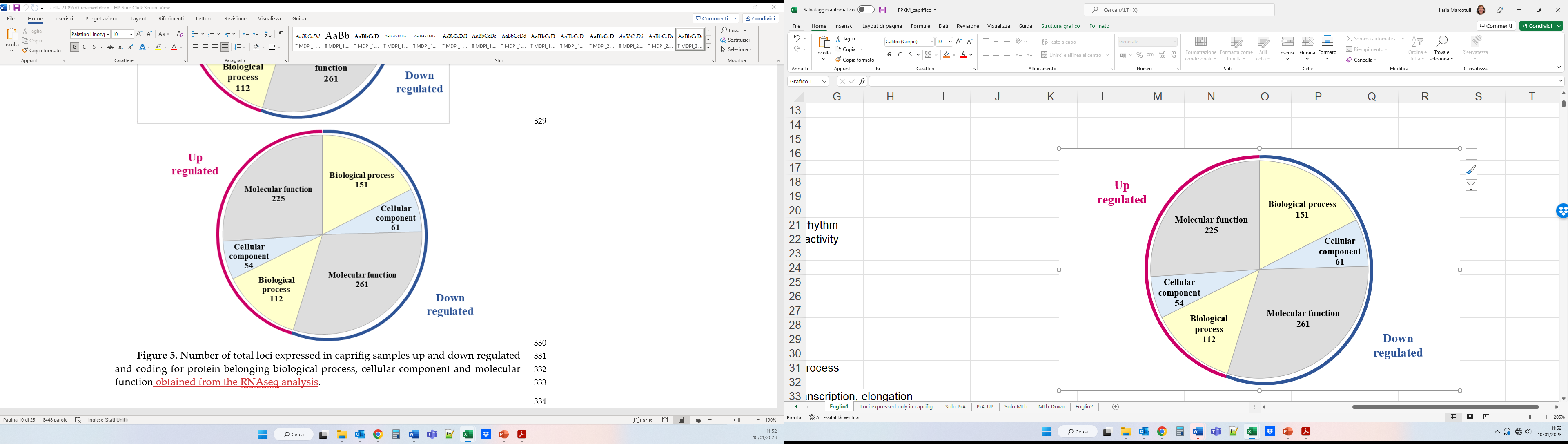


**Figure S2.** Number of total loci expressed in caprifig samples up and down regulated and coding for protein belonging biological process, cellular component and molecular function obtained from the RNAseq analysis.


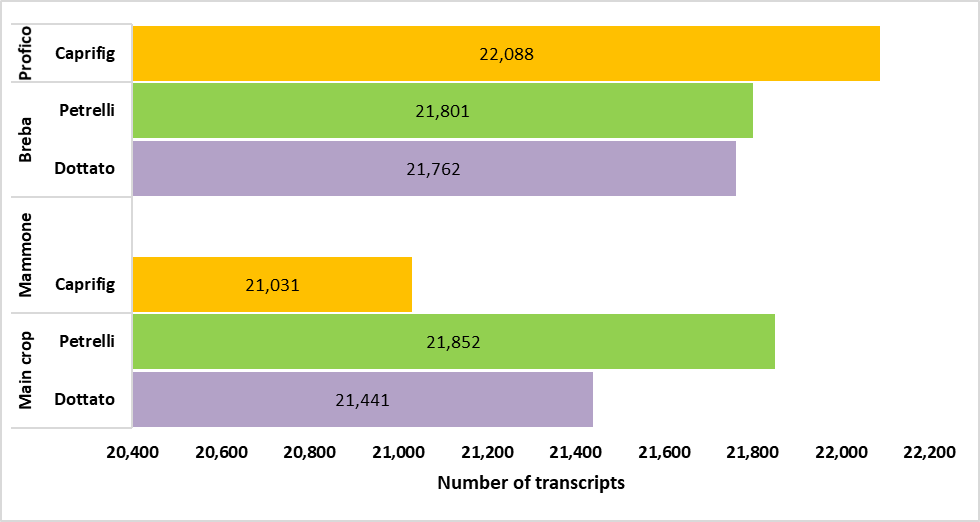


**Figure S3.** Number of total loci expressed in caprifig (orange color), ‘Petrelli’ (green color) and ‘Dottato’ (purple color) samples up and down regulated and coding for protein belonging biological process, cellular component and molecular function obtained from the RNAseq analysis.
